# Juxta-Vascular Pulmonary Nodule Segmentation in PET-CT Imaging Based on an LBF Active Contour Model with Information Entropy and Joint Vector

**DOI:** 10.1155/2018/2183847

**Published:** 2018-01-08

**Authors:** Rui Hao, Yan Qiang, Xiaofei Yan

**Affiliations:** ^1^College of Information Management, Shanxi University of Finance & Economics, Taiyuan 030006, China; ^2^College of Computer Science and Technology, Taiyuan University of Technology, Taiyuan 030024, China

## Abstract

The accurate segmentation of pulmonary nodules is an important preprocessing step in computer-aided diagnoses of lung cancers. However, the existing segmentation methods may cause the problem of edge leakage and cannot segment juxta-vascular pulmonary nodules accurately. To address this problem, a novel automatic segmentation method based on an LBF active contour model with information entropy and joint vector is proposed in this paper. Our method extracts the interest area of pulmonary nodules by a standard uptake value (SUV) in Positron Emission Tomography (PET) images, and automatic threshold iteration is used to construct an initial contour roughly. The SUV information entropy and the gray-value joint vector of Positron Emission Tomography–Computed Tomography (PET-CT) images are calculated to drive the evolution of contour curve. At the edge of pulmonary nodules, evolution will be stopped and accurate results of pulmonary nodule segmentation can be obtained. Experimental results show that our method can achieve 92.35% average dice similarity coefficient, 2.19 mm Hausdorff distance, and 3.33% false positive with the manual segmentation results. Compared with the existing methods, our proposed method that segments juxta-vascular pulmonary nodules in PET-CT images is more accurate and efficient.

## 1. Introduction

Lung cancer is a crucial threat to human life in recent years [1]. Peripheral lung cancers are the most common type of lung cancers. Juxta-vascular nodules are the main symptom in the early stages of peripheral lung cancers. Due to the abundance of nutrients in the blood vessels, they are provided with a good foundation for growth. Therefore, juxta-vascular nodules have the great probability of being malignant nodules. The early detection and diagnose of juxta-vascular nodules have important significance to treat the lung cancer which can immensely improve the survival rates of patients. Currently, computer-aided diagnosis (CAD) systems can analyze a large number of nodule medical images automatically which are effective tools to improve the accuracy of pulmonary nodule diagnosis. Accurate juxta-vascular nodule segmentation is the prerequisite for nodule detection and benign/malignant diagnosis by the CADs [2, 3]. However, due to vascular interference, it is easy to cause the oversegmentation for juxta-vascular nodules.

Recently, a lot of researches have been done on the segmentation of juxta-vascular pulmonary nodules, and various methods have been proposed. Chen et al. [4] presented a segmentation method for vessel attachment nodules based on integrated active contour model, which can effectively solve the problem of nodules with similar intensity and intensity inhomogeneity, but this algorithm performance depends largely on the choice of the initial position. Farag et al. [5] proposed a variational level set approach for lung nodule segmentation fusing with the image intensity statistical information in a shape model. This technique does not depend on the nodule types or locations, but the segmentation performance for juxta-vascular nodules is ineffective. Mukhopadhyay [6] proposed a segmentation framework for all types of pulmonary nodules. In the framework, pulmonary nodules are classified into solid/part-solid and nonsolid categories by analyzing intensity distribution in the core of the nodules. After determining the categories of the nodules, the particular algorithm is set to remove blood vessels from the nodule body by vasculature pruning technique. The classification accuracy is crucial for segmentation performance. Qi et al. [7] used a two-dimensional ray casting and linear fitting method to segment the juxta-vascular pulmonary nodules, whose algorithm required users to select and determine an optimal area as the seed points for segmentation, so it is difficult to guarantee the reproducibility of a segmentation result. Sun et al. [8] proposed a method for segmentation of juxta-vascular pulmonary nodules based on flow entropy and geodesic distance. The average segmentation accuracy rate reached 91.77%, but the reproducibility of results was still unsatisfactory. Si et al. [9] proposed a new segmentation method to deal with the juxta-vascular pulmonary nodules which used a 3D ray casting method to extract the surface information of the nodules. Meanwhile, this method adopted the 3D distance transformation method to improve the reproducibility of the segmentation results and minimized influence of selecting seed points manually. The segmentation accuracy exceeded 90%.

Although researchers have proposed many segmentation methods of juxta-vascular pulmonary nodules, most of these methods require manual interaction and make a seed point manually. Thus, it is difficult to realize the nodule segmentation automatically. Moreover, there is high gray-level similarity between nodules and attached blood vessels in the CT images. The circular area of vascular cross-section also has a great disturbance for the nodule segmentation. How to segment the juxta-vascular nodules automatically and accurately is still a challenging problem.

Active contour model is one of the most popular segmentation methods in recent years. To achieve accurate segmentation, a contour curve is driven to evolve toward the energy reduction direction by minimizing the energy functional. This method has been widely applied in the field of medical image segmentation and greatly reduces the possibility of the edge leakage [10–12]. The local binary fitting (LBF) model, as one of the effective active contour models, is used to fit the local regions gray information by a Gauss kernel function to improve the segmentation results of the CT images with noise and intensity inhomogeneity [13]. However, in the LBF model, it cannot get the precise pulmonary nodules edge only with the gray features of the CT images, especially for juxta-vascular nodules. PET-CT is a diagnostic method to combine metabolic function imaging and anatomical structure imaging [14]. In PET images, SUV can accurately reflect the metabolic information of lesion tissues. SUV can be used to eliminate the influence of vessels and other tissues to the greatest extent and achieve accurate analysis of the pulmonary nodules.

In this paper, we propose an automatic juxta-vascular nodule segmentation method. The multiple features in PET-CT images based on an LBF active contour model are used to drive the evolution of a contour curve to stop at the edge of the pulmonary nodule accurately. Experimental results show that our method can segment the juxta-vascular nodules efficiently and accurately.

The rest of the paper is organized as follows.  Section 2 presents the PET-CT imaging that was employed to validate the proposed methods.  Section 3 presents our novel segmentation model for juxta-vascular nodules in detail.  Section 4 shows the performance metric for evaluation of results.  Section 5 shows the experimental results, comparisons with other segmentation methods, and discussion. The conclusion is presented in Section 6.

## 2. Materials

The PET-CT imaging data was obtained from a hospital in Shanxi, China, and was produced by Discovery ST16 PET/CT (GE Healthcare, USA) with the parameters of 140 kV, 150 mA, and slice thickness 3.75 mm. The size of the CT images is 512 × 512 while that of the PET images is 128 × 128. Because the different resolutions between them, PET images were coregistered to the CT images using rigid transformation based on the Elastix toolbox [15]. After the registration, we can obtain the one-to-one voxel correspondence between the PET and CT images.

The experiment used PET-CT images of 392 patients. There are 299 PET and 299 CT images for each patient. Among these images, we chose 814 juxta-vascular nodules PET-CT images for experiment (the maximum diameter ranged from 1.3 to 24 mm, with an average maximum diameter of 6.1 mm). Regarding patient privacy, we signed the relevant confidentiality agreement with the hospital and patients, and the corresponding processing had been done for patient's information on PET-CT images.

## 3. Methods

### 3.1. Construction of Initial Contour

The construction of the initial contour is the first step in active contour model and usually requires manual annotation. However, because the pulmonary nodules are smaller and vague in the CT images, it is difficult for physicians to identify and mark the pulmonary nodules accurately with naked eyes. Therefore, in this paper, multiple features in the PET-CT images are used to construct the initial contour of pulmonary nodules automatically.

#### 3.1.1. Extraction of Pulmonary Nodule Region of Interests (ROIs)

**Figure 1:**
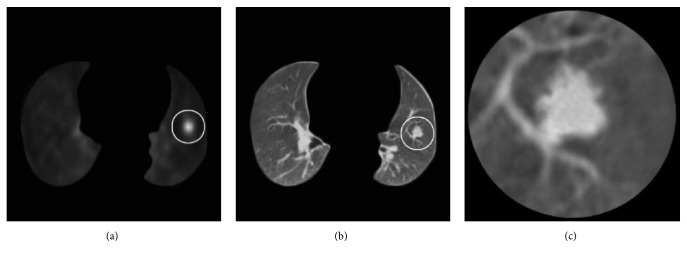
ROIs extraction of the pulmonary nodule. (a) Location of the nodule in PET image; (b) location of the nodule in CT image corresponding to PET image (a); (c) ROI of the pulmonary nodule.

To narrow down the range of segmentation, it is necessary to extract the ROIs of the pulmonary nodule before the construction of the initial contour. First, the Otsu threshold algorithm and the morphological open operation are used to segment the lung parenchyma [16]. In PET images, the metabolism of the nodule region is relatively active, and corresponding SUV is higher than other nonlesion regions. According to this characteristic, we can get the pixel *O*, the SUV value of which is the maximum in lung parenchyma, and construct a circular template with the center *O* and radius *R* in the PET images. Then, it is registered to the CT image as the pulmonary nodule ROIs. In general, the diameter of the pulmonary nodule is in range of 3 mm to 30 mm. To avoid the omission of some lesion regions, we set *R* = 30 mm.  Figure 1 shows the results of ROIs.

#### 3.1.2. Automatic Threshold Iteration

There is no need for a high degree of accuracy in the construction of the initial contour of the pulmonary nodule; instead, there is need for high execution efficiency. The threshold segmentation algorithm can roughly determine the initial contour of pulmonary nodules with the higher segmentation speed. The main idea of the threshold segmentation is that users have to set fixed gray values before segmenting. However, it is hard to choose a suitable global threshold manually to get the optimal initial contour due to the differences of gray values in the different pulmonary nodule CT images. Thus, in this paper, automatic threshold iterative algorithm is adopted to construct the initial contour. The description of steps for initial contour is given as follows.

**Step 1 .**

Set the initial threshold value *T* according to

(1) $$\begin{array}{c} T=\frac{\left(G_{max}+G_{min}\right)}{2}, \end{array}$$

where *G*_max_ and *G*_min_ represent the maximum and minimum gray values in the CT images, respectively.

**Step 2 .**

Divide the entire ROIs into two sets of pixels using *T*: the set *B* (background region) and *N* (nodule region).

**Step 3 .**

Calculate the average gray values *α*_*b*_ in set *B* and *α*_*n*_ in set *N*.

**Step 4 .**

Update the new threshold according to

(2) $$\begin{array}{c} T=\frac{\alpha_{b}+\alpha_{n}}{2}. \end{array}$$

**Step 5 .**

Repeat Steps 2–4 until |*T*_*n*_ − *T*_*n*−1_| ≤ *λ*, where *T*_*n*−1_ is the threshold at iteration *n* − 1 and *T*_*n*_ represents the threshold at iteration *n*. *λ* is the preset parameter.

After several iterations, optimal gray threshold value *T* is obtained, and the operation of binarization in the CT images can be done according to

(3) $$\begin{array}{c} I_{bin}\left(x,y\right)=\left\{\begin{array}{cc} 1, & I\left(x,y\right)\geq T\\ 0, & I\left(x,y\right)<T, \end{array}\right. \end{array}$$

where *I*_bin_(*x*, *y*) denotes the binary CT images.

**Figure 2:**
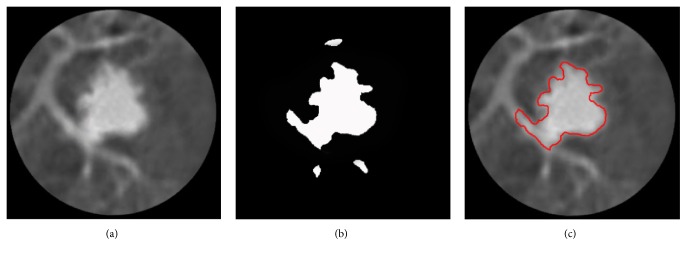
The result of initial contour. (a) ROI of pulmonary nodule; (b) binary CT image; (c) initial contour.

After the above operations, we cannot get the initial contour of pulmonary nodule due to some noise spots or complicated tissue in the ROIs. Therefore, we detect the edges of the binary CT images and calculate the area enclosed by these edges. Finally, the edge of the maximum area is selected as the optimal initial contour. The result of the initial contour is shown in Figure 2.

### 3.2. LBF Active Contour Model Based on Information Entropy and Joint Vector

#### 3.2.1. LBF Active Contour Model

Li et al. proposed the LBF active contour model to overcome the poor image segmentation with noise and intensity inhomogeneity [13]. In the LBF model, the energy functional is redefined by the variable local fitting energy. Let the initial contour *C* divide the image domain *I* into two regions: *Ω*_1_ and *Ω*_2_, where *Ω*_1_ denotes the inside region of *C* and *Ω*_2_ denotes the outside region of *C*. For image *I*, local fitting energy functional of each pixel is formulated as follows:

(4) $$\begin{array}{c} E_{x}\left(C,f_{1}\left(x\right),f_{2}\left(x\right)\right)=\lambda_{1}\overset{}{\underset{\Omega_{1}}{\int }}K_{\sigma }\left(x-y\right){\left|I\left(y\right)-f_{1}\left(x\right)\right|}^{2}dy+\lambda_{2}\overset{}{\underset{\Omega_{2}}{\int }}K_{\sigma }\left(x-y\right){\left|I\left(y\right)-f_{2}\left(x\right)\right|}^{2}dy, \end{array}$$

where *λ*_1_ and *λ*_2_ are the weighted coefficients that balance the energy between two regions. *f*_1_(*x*) and *f*_2_(*x*) represent the local gray fitting values of inside and outside initial contour, respectively. They are determined by the set of pixels *y* which is in the neighborhood of pixel *x*. *K*_*σ*_(*x* − *y*) denotes the Gauss kernel function with the standard deviation *σ*.

*K*_*σ*_(*x* − *y*) can be formulated as follows:

(5) $$\begin{array}{c} K_{\sigma }\left(x-y\right)=\frac{1}{\sqrt{2\pi }\sigma }\exp\left(-\frac{{\left(x-y\right)}^{2}}{2\sigma^{2}}\right). \end{array}$$

The LBF model uses the Gaussian kernel function *K*_*σ*_(*x* − *y*). Thus, it can deal with intensity inhomogeneity very well. However, the LBF model only considers the distance relationship between the different pixels and ignores the information of the pixel itself by the Gaussian kernel function. Thus, the LBF model may cause the problem of edge leakage especially for the segmentation of juxta-vascular nodules. In addition, some boundary regions between the pulmonary nodules and the pulmonary parenchyma may be blurred and the contrast is relatively low. It cannot get the precise pulmonary nodule edge only using the gray features of the CT images. Therefore, we improve the LBF active contour model by combining the multiple features in PET-CT images to achieve the accurate segmentation of juxta-vascular nodules.

#### 3.2.2. Edge Guide Function of Information Entropy

To overcome the edge leakage in segmentation of juxta-vascular nodules, we use an edge guide function based on information entropy to drive the evolving contour curve, which will stop at the edges of pulmonary nodules accurately.

In 1948, Shannon introduced the concept of entropy into information theory from thermodynamics and proposed the information entropy theory for the purpose of quantification of uncertain information in systems [17]. Assuming that an information system is composed of *n* random variables {*x*_1_, *x*_2_,…, *x*_*n*_} and the probability of each random variable *x*_*i*_ is *p*_*i*_, the information entropy of this system can be formulated as

(6) $$\begin{array}{c} H=-\overset{n}{\underset{i=1}{\sum }}p_{i}\log_{2}p_{i}. \end{array}$$

Formula (6) indicates that information entropy *H* is the maximum value if the probability distribution of the random variables is equal.

In PET images, the metabolism of pulmonary nodules is more active than that of vessels, and SUV value of pulmonary nodules is higher than that of vessels. The distribution of SUV value between nodules and vessels has a greater difference. Motivated by this characteristic, we present the SUV information entropy to drive the evolving contour curve.

**Figure 3:**
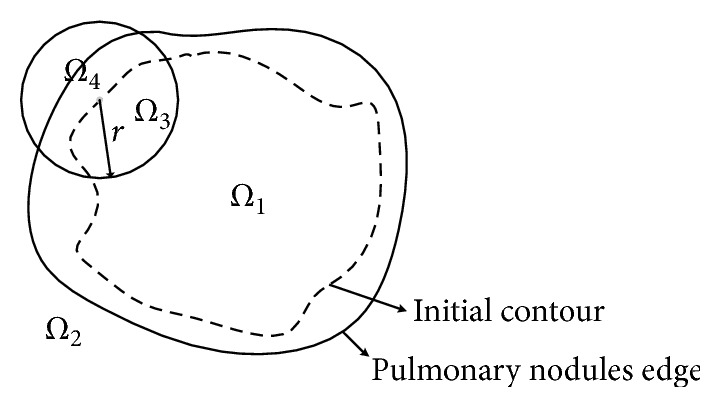
Schematic diagram of region.

Given a point *x* on the initial contour curve, *R*(*x*) represents a circular region with the center point *x* and the radius *r* pixels, as shown in Figure 3. *R*(*x*) is partitioned into two parts, *Ω*_3_ and *Ω*_4_, by the initial contour curve. SUV information entropy in region *Ω*_3_ and *Ω*_4_ is formulated as follows:

(7) $$\begin{array}{c} H_{SUV}^{i}=-\overset{}{\underset{R\left(x\right)}{\iint }}P_{i}\left(x\right)\log_{2}P_{i}\left(x\right), \end{array}$$

where *P*_*i*_(*x*) represents the SUV probability distribution value in region *Ω*_3_ and *Ω*_4_. Because the area of the pulmonary nodule is small, the probability of SUV is equal to the Gauss distribution approximately. Therefore, *P*_*i*_(*x*) is formulated as follows:

(8) $$\begin{array}{c} P_{i}\left(x\right)=\frac{1}{\sqrt{2\pi }\sigma_{i}\left(x\right)}\exp\left(-\frac{{\left(I_{i}\left(x\right)-c_{i}\left(x\right)\right)}^{2}}{2\sigma_{i}^{2}\left(x\right)}\right), \end{array}$$

where *I*_*i*_(*x*) is the SUV value of pixel *x*. *c*_*i*_(*x*) and *σ*_*i*_(*x*) represent the mean and standard deviation of SUV value in region *Ω*_3_ and *Ω*_4_, respectively.

Further, we define a characteristic function in terms of *R*(*x*) and formulate it as follows:

(9) $$\begin{array}{c} \varphi \left(x,y\right)=\left\{\begin{array}{cc} 1, & y\in R\left(x\right)\\ 0, & y\notin R\left(x\right) \end{array}\right.. \end{array}$$

Thus, the edge guide function based on SUV information entropy can be written as

(10) $$\begin{array}{c} F_{i}\left(x\right)=e^{-\varphi \left(x,y\right)H_{SUV}^{i}}. \end{array}$$

For the regions *Ω*_3_ and *Ω*_4_, if the SUV distribution is more homogeneous in one of them, the information entropy is bigger and the corresponding edge guide function is smaller. Therefore, the evolving contour curve should gradually stop at the boundaries of this region. At this moment, the target and the background can be segmented well. On the contrary, it indicates that this region contains both the target and the background and the curve should continue to deform and displace until the SUV information entropy in region *Ω*_3_ and *Ω*_4_ does not change. For the juxta-vascular nodules, there is a larger difference in SUV value between nodules and vessels. The distribution of SUV is inhomogeneous in the boundary region of juxta-vascular nodules; thus the contour curve can evolve continuously and accurately with the guidance of the edge guide function.

#### 3.2.3. Energy Functional

The LBF model neglects the information of the pixel itself by the Gaussian kernel function. Thus, we replace the Gaussian kernel function by an edge guide function based on SUV information entropy. In addition, the contrast may be lower between pulmonary nodules and pulmonary parenchyma. It is difficult to locate the edge of the pulmonary nodule accurately only using the gray values in the CT image. In PET images, the gray value of nodules is higher than that of other nonlesion tissues. According to this, we define a joint vector **f** = (*f*_1_, *f*_2_)^*T*^ by combining the gray values in CT and PET images, where *f*_1_ and *f*_2_ represent the Gaussian gray fitting values of the CT image and the PET image, respectively. Therefore, the energy functional of an improved LBF model is formulated as follows:

(11) $$\begin{array}{c} E_{x}^{\prime }\left(C,f_{1}\left(x\right),f_{2}\left(x\right)\right)=\lambda_{1}\overset{}{\underset{\Omega_{1}}{\int }}F_{1}\left(x\right)\Lambda {\left|I\left(y\right)-f_{1}\left(x\right)\right|}^{2}dy+\lambda_{2}\overset{}{\underset{\Omega_{2}}{\int }}F_{2}\left(x\right)\Lambda {\left|I\left(y\right)-f_{2}\left(x\right)\right|}^{2}dy, \end{array}$$

where **f**_1_(*x*) and **f**_2_(*x*) represent the Gaussian gray fitting values of the joint vector in region of *Ω*_1_ and *Ω*_2_. We set *λ*_1_ = *λ*_2_ = 1. The standard deviation of the Gaussian kernel function is *σ*. Λ is the coefficient matrix and is defined in (12). In this paper, we set Λ_1_ = Λ_2_ = 1.

(12) $$\begin{array}{c} \Lambda =\left(\begin{array}{cc} \Lambda_{1} & 0\\ 0 & \Lambda_{2} \end{array}\right). \end{array}$$

#### 3.2.4. The Level Set Formulation

The level set function *ϕ* introduced by Osher and Sethian [18] is an efficient numerical method for solving curve evolution. Curve *C* can be represented by the zero level set. With the level set representation, the energy functional in (11) can be reformulated as follows:

(13) $$\begin{array}{c} E_{x}^{\prime }\left(\phi ,f_{1}\left(x\right),f_{2}\left(x\right)\right)=\lambda_{1}\overset{}{\underset{\Omega_{1}}{\int }}F_{1}\left(x\right)\Lambda {\left|I\left(y\right)-f_{1}\left(x\right)\right|}^{2}H\left(\phi \left(y\right)\right)dy+\lambda_{2}\overset{}{\underset{\Omega_{2}}{\int }}F_{2}\left(x\right)\Lambda {\left|I\left(y\right)-f_{2}\left(x\right)\right|}^{2}\left(1-H\left(\phi \left(y\right)\right)\right)dy, \end{array}$$

where *H*(*x*) is the function of Heaviside and the level function *ϕ* is defined as follows:

(14) $$\begin{array}{c} \phi \left(x\right)>0,\;x\in \Omega_{1}\\ \phi \left(x\right)=0,\;x\in C\\ \phi \left(x\right)<0,\;x\in \Omega_{2}. \end{array}$$

In practice, it is approximated by a smooth function *H*_*ε*_(*x*), which is formulated as follows:

(15) $$\begin{array}{c} H_{\varepsilon }\left(x\right)=\frac{1}{2}\left[1+\frac{2}{\pi }\arctan\left(\frac{x}{\varepsilon }\right)\right], \end{array}$$

where *ε* is a positive constant and used to control the rising rate of Heaviside function from 0 to 1. We set *ε* = 1. Thus, the fitting energy terms in (13) can be reformulated as

(16) $$\begin{array}{c} E^{\prime }=\overset{}{\underset{\Omega }{\int }}E_{x}^{\prime }\left(\phi ,f_{1}\left(x\right),f_{2}\left(x\right)\right)dx. \end{array}$$

To maintain the smoothness of the evolving curve during the segmentation process, we introduce the length term of zero level curve [19]:

(17) $$\begin{array}{c} L\left(\phi \right)=\overset{}{\underset{\Omega }{\int }}\delta \left(\phi \left(x\right)\right)\left|\nabla \phi \left(x\right)\right|dx, \end{array}$$

where *δ*(*x*) is called Dirac function and is the derivative of *H*_*ε*_(*x*). It is formulated as

(18) $$\begin{array}{c} \delta \left(x\right)=\frac{d}{dx}H_{\varepsilon }\left(x\right)=\frac{1}{\pi }\frac{\varepsilon }{\varepsilon^{2}+x^{2}}. \end{array}$$

After several iterations, the evolution of the level set function may be unstable and the result is likely to be inaccurate. It needs reinitializing the level set function in a certain period of time. Therefore, we add the distance regularizing term into (16) to penalize the deviation of the level set function from a signed distance function [19]. The distance regularizing term is

(19) $$\begin{array}{c} P\left(\phi \right)=\overset{}{\underset{\Omega }{\int }}\frac{1}{2}{\left(\left|\nabla \phi \left(x\right)\right|-1\right)}^{2}dx. \end{array}$$

Thus, we define the entire level set energy functional as follows:

(20) $$\begin{array}{c} F=E^{\prime }+\nu L\left(\phi \right)+\mu P\left(\phi \right), \end{array}$$

where *ν* and *μ* are the positive constants and denote the coefficients of the length penalty term and the distance regularizing term, respectively.

The optimal segmentation result is usually the process of solving the minimum of the energy functional. Thus, the energy functional in (20) can be minimized by using the gradient descent flow equations. It is formulated as follows:

(21) $$\begin{array}{c} \frac{\partial \phi }{\partial t}=-\delta \left(\phi \right)\left(\lambda_{1}e_{1}+\lambda_{2}e_{2}\right)+\nu \delta \left(\phi \right)div\left(\frac{\nabla \phi }{\left|\nabla \phi \right|}\right)+\mu \left(\nabla^{2}\phi -div\left(\frac{\nabla \phi }{\left|\nabla \phi \right|}\right)\right), \end{array}$$

where *e*_1_ and *e*_2_ are defined as formula (22). **f**_1_(*x*) and **f**_2_(*x*) are defined as formula (23).

(22) $$\begin{array}{c} e_{1}=\overset{}{\underset{\Omega_{1}}{\int }}F_{1}\left(x\right)\Lambda {\left|I\left(y\right)-f_{1}\left(x\right)\right|}^{2}H\left(\phi \left(y\right)\right)dy\\ e_{2}=\overset{}{\underset{\Omega_{2}}{\int }}F_{2}\left(x\right)\Lambda {\left|I\left(y\right)-f_{2}\left(x\right)\right|}^{2}\left(1-H\left(\phi \left(y\right)\right)\right)dy \end{array}$$

(23) $$\begin{array}{c} f_{1}\left(x\right)=\frac{\int_{\Omega }K_{\sigma }\left(x-y\right)I\left(y\right)H_{\varepsilon }\left(\phi \left(y\right)\right)dy}{\int_{\Omega }K_{\sigma }\left(x-y\right)H_{\varepsilon }\left(\phi \left(y\right)\right)dy}\\ f_{2}\left(x\right)=\frac{\int_{\Omega }K_{\sigma }\left(x-y\right)I\left(y\right)\left(1-H_{\varepsilon }\left(\phi \left(y\right)\right)\right)dy}{\int_{\Omega }K_{\sigma }\left(x-y\right)\left(1-H_{\varepsilon }\left(\phi \left(y\right)\right)\right)dy}. \end{array}$$

The description of steps of our proposed method based on information and joint vector is given as follows.

**Step 1 .**

Set the initial level set function *ϕ* = 0 and the counter *k* = 0.

**Step 2 .**

Calculate the edge guide function *F*_1_(*x*) and *F*_2_(*x*) according to (10), and calculate the gray fitting values of joint vector **f**_1_(*x*) and **f**_2_(*x*) according to (23).

**Step 3 .**

Calculate the level set energy functional *F*, and set *k* = *k* + 1.

**Step 4 .**

Update the level set function according to (21).

**Step 5 .**

Repeat Steps 2–4 until any one of the following conditions is met:

1. The level set energy functional is stable; that is, Δ*F* = *F*_*k*_–*F*_*k*−1_ ≤ *χ*.
2. *k* reaches the maximum number of iterations *K*_max_,; that is, *k* ≤ *K*_max_.

## 4. Performance Metric for Evaluation of Results

To demonstrate the superiority of our method objectively, three metrics (*dice similarity coefficient* [20],* Hausdorff distance* [6],* and false positive* [21]) are used to evaluate the performances of our method.

It is difficult to get each patient's pathological samples as the ground truth for segmentation of pulmonary nodules. Thus, in experiment, we invited an expert with rich experience to do manual segmentation for pulmonary nodules. The results were used as the ground truth.

### 4.1. Dice Similarity Coefficient

Dice similarity coefficient (DSC) is a measure to evaluate the overlap ratio between ground truth *A*_*g*_ and actual segmentation results *A*_*r*_. DSC can be computed as follows:

(24) $$\begin{array}{c} DSC\left(A_{g},A_{r}\right)=\frac{2\times \left(A_{g}\cap A_{r}\right)}{\left|A_{g}\right|+\left|A_{r}\right|}\times 100. \end{array}$$

The higher the value of Dice similarity coefficient is, the better the performance of nodules segmentation algorithm is.

### 4.2. Hausdorff Distance

Hausdorff distance (HD) is a parameter to evaluate the shortest distance between ground truth and actual segmentation boundaries, which can reflect the coincidence degree of two images.

Let *A*_*g*_ = {*a*_*g*1_, *a*_*g*2_,…, *a*_*gm*_} represent the contour pixels set of the ground truth and *B*_*r*_ = {*b*_*r*1_, *b*_*r*2_,…, *b*_*rm*_} represent the contour pixels set of the actual segmentation algorithm. HD can be computed as follows:

(25) $$\begin{array}{c} HD\left(A_{g},B_{r}\right)=\max\left(h\left(A_{g},B_{r}\right),h\left(B_{r},A_{g}\right)\right)\\ h\left(A_{g},B_{r}\right)=\underset{a_{g}\in A_{g}}{\max}\;\underset{b_{r}\in B_{r}}{\min}\left\|a_{g}-b_{r}\right\|, \end{array}$$

where ‖*a*_*g*_ − *b*_*r*_‖ is the Euclidean distance between the pixel *a*_*g*_ and *b*_*r*_. The smaller the HD is, the better the segmentation results are.

### 4.3. False Positive

False positive (FP) denotes the number of false positive pixels, which is marked as the nodule in actual results but not in ground truth results. It can be computed as follows:

(26) $$\begin{array}{c} FP=\frac{\left|A_{r}-A_{g}\cap A_{r}\right|}{\left|A_{g}\right|}\times 100. \end{array}$$

The smaller the FP is, the better the accuracy of segmentation algorithm is.

## 5. Results and Discussion

In this section, we compare the segmentation results of our method with some existing algorithms. All of experiments were implemented in Matlab 2012b and executed on a personal computer equipped with 8 GB RAM and a 2.53 GHz Intel Core i5-3770 processor.

To evaluate the performances of our method for segmentation of juxta-vascular nodules, we compared our method with the existing algorithms, including the LBF active contour model [13], the vasculature pruning technique (VP) [6], the 2D ray casting and linear fitting algorithm (RCLF) [7], and the flow entropy and geodesic distance method (FEGD) [8]. The results of ground truth are used as the standard for the performances of the above algorithms.

### 5.1. Parameters Setting

We need to set the appropriate parameters to ensure the efficiency and accuracy of the algorithm. There are 7 important parameters. *λ* is the number of difference between two thresholds. It will affect the position of initial contour. *r* represents the number of radius pixels. *σ* is the standard deviation of the Gaussian kernel function. *ν* and *μ* denote the coefficients of the length penalty term and the distance regularizing term, respectively. *χ* represents the difference between energy functional *F*_*k*−1_ and *F*_*k*_. *K*_max_ denotes the maximum number of iterations of the program. These parameters were optimized through a large number of experiments which can ensure both accurate results and lower time complexity.  Table 1 lists the parameters and the values used in our method.

### 5.2. Results of Segmentation

We applied the proposed segmentation method to 400 juxta-vascular nodules for testing.

**Figure 4:**
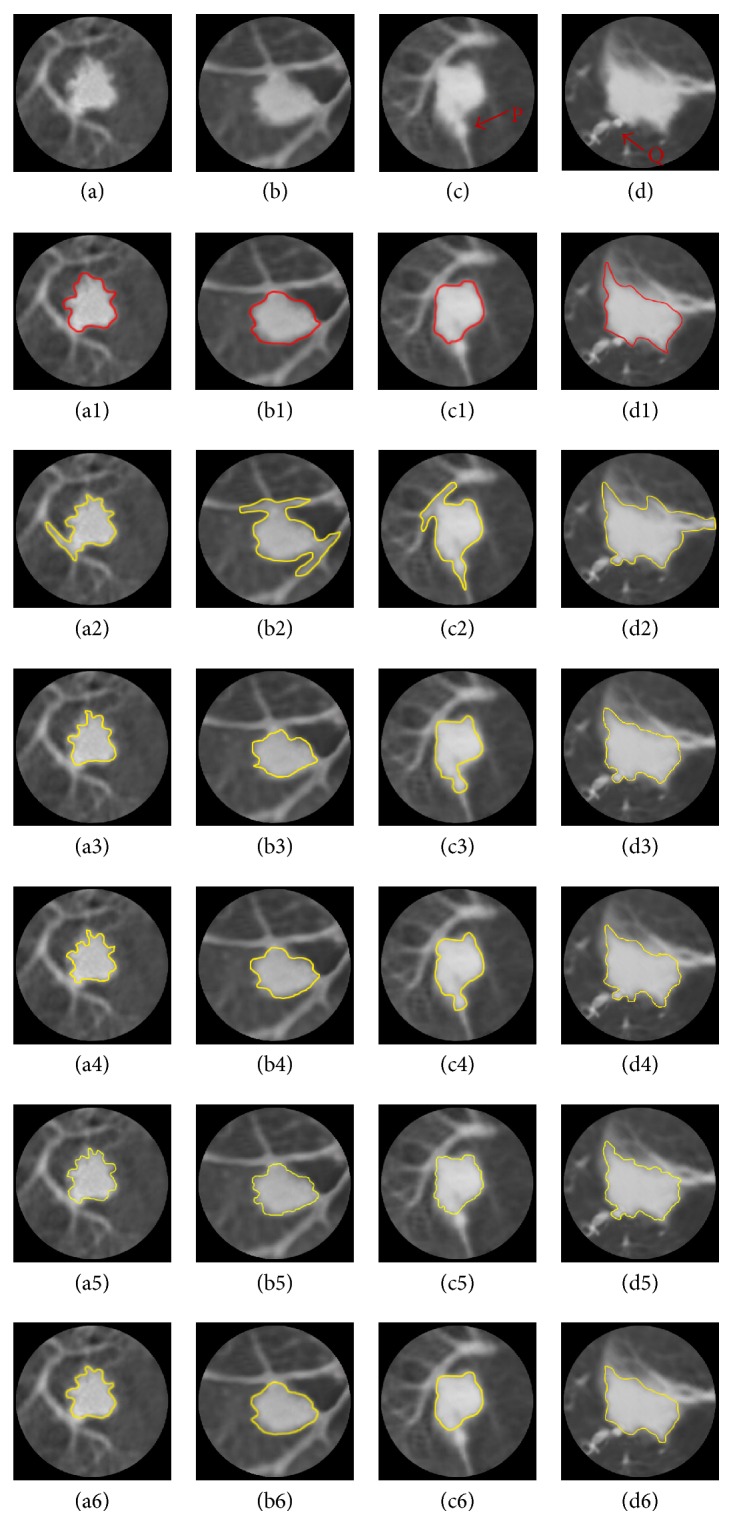
Comparison of segmentation results by different algorithms for juxta-vascular nodules.

Figure 4 shows the segmentation results of the juxta-vascular nodules segmentation. The red curve is the result of the ground truth; the yellow curve is the result of the different algorithms for juxta-vascular nodules. In Figure 4, the first row contains 4 original ROI images of juxta-vascular nodules (a–d). In (c) and (d) images, P and Q are the areas of vascular cross-section; the second row is the results of the ground truth (a1–d1); the third row is the results by the LBF model (a2–d2); the fourth row is the results by the RCLF algorithm (a3–d3); the fifth row is the results by the FEGD algorithm (a4–d4); the sixth row is the results by the VP algorithm (a5–d5); and the last row is the results by our method (a6–d6).

As shown in (a2), (b2), (c2), and (d2) of Figure 4, there is severe edge leakage by the LBF model and the vessel is not separated from nodules.

For nodule (a) and (b), the results of our method (Figure 4, (a6) and (b6)), RCLF (Figure 4, (a3) and (b3)), FEGD (Figure 4, (a4) and (b4)), and VP (Figure 4, a(5) and b(5)) are consistent with the ground truth (Figure 4, (a1) and (b1)). However, for nodule (c) and (d), there is certain edge leakage by the RCLF and FEGD. And VP cannot segment the area Q. The reason is that methods of RCLF, FEGD, and VP cannot deal with the circular area of vascular cross-section very well.

Last row in Figure 4 shows that the results of the proposed method are more consistent with the ground truth and can segment the juxta-vascular nodules accurately, especially for nodule (c) and (d). Meanwhile, the edge of the proposed method is smoother than other results.

### 5.3. Quantitative Comparisons

**Figure 5:**
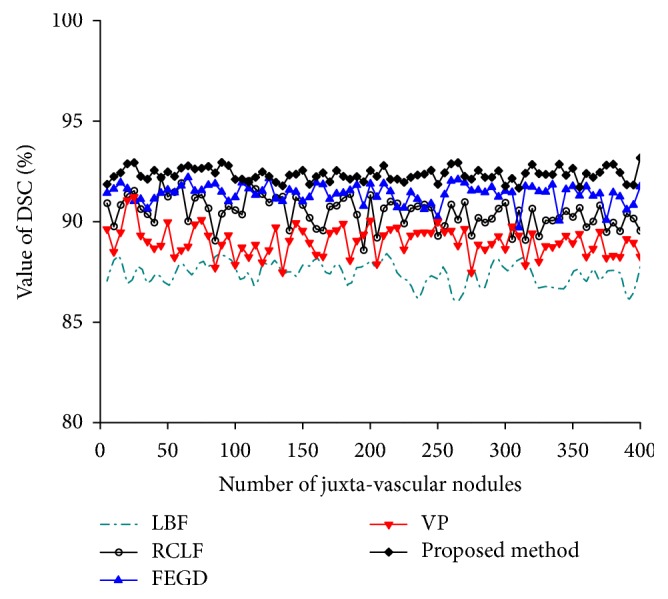
The DSC values of the five methods on the segmentation results of pulmonary nodules.

Figure 5 shows the DSC values of five methods for the segmentation of 400 images of juxta-vascular nodules.

**Figure 6:**
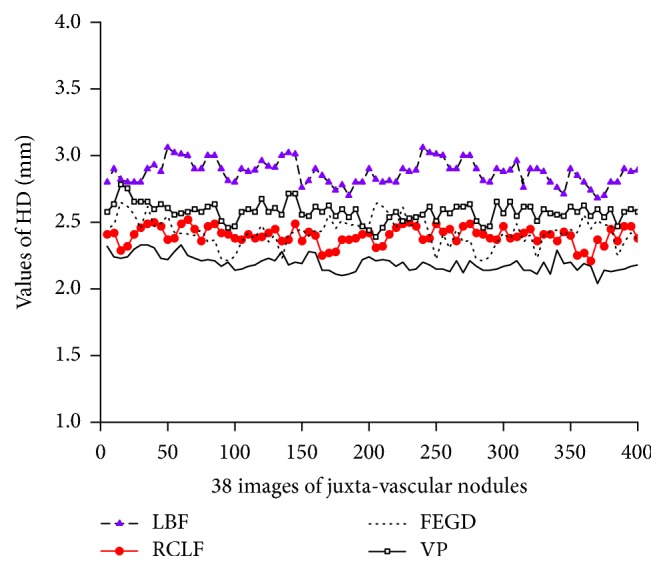
The HD values of five methods on the segmentation results of pulmonary nodules. The black solid line at the bottom refers to the proposed method.

Figure 6 shows the HD values of five methods for the segmentation of 400 images of juxta-vascular nodules.

**Figure 7:**
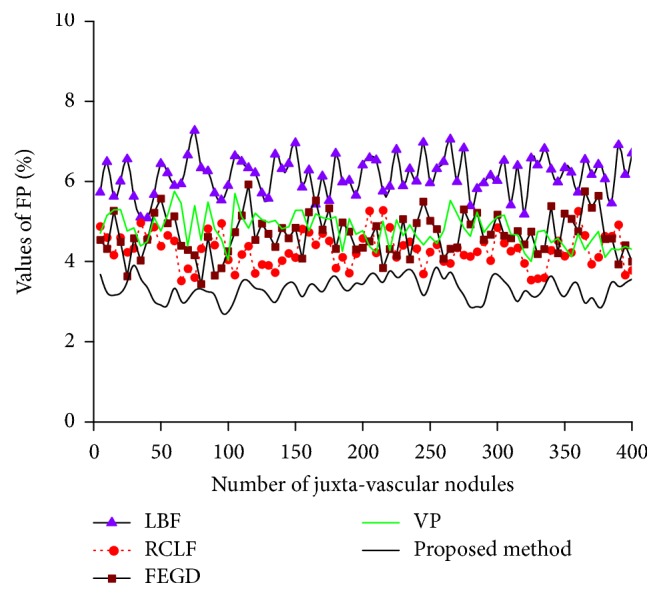
The FP values of the five methods on the segmentation results of pulmonary nodules.

Figure 7 shows the FP values of five methods for the segmentation of 400 images of juxta-vascular nodules.

It is clear from Figure 5 that FEGD algorithm is close to our method which is better than the LBF, RCLF, and VP algorithms in terms of the DSC value. For 400 images of juxta-vascular nodules, the DSC value of our method varies less than those of the other four algorithms. The change of HD value of our method is relatively stable, which is slightly lower than that of RCLF algorithm in Figure 6. Figure 7 shows that our method is the global minimum in terms of the FP value.

Table 2 illustrates the average values of DSC, Hausdorff, and FP by our method and other algorithms. It is clear from Table 2 that our method performs better than LBF, RCLF, FEGD, and VP algorithms in terms of the average values of DSC, Hausdorff, and FP.

In general, based on the analysis of the three metrics DSC, Hausdorff, and FP, the segmentation results of our method are more consistent with the ground truth results, which can further reflect the high preformation and stability of our method for the segmentation of test images of juxta-vascular nodules.

**Figure 8:**
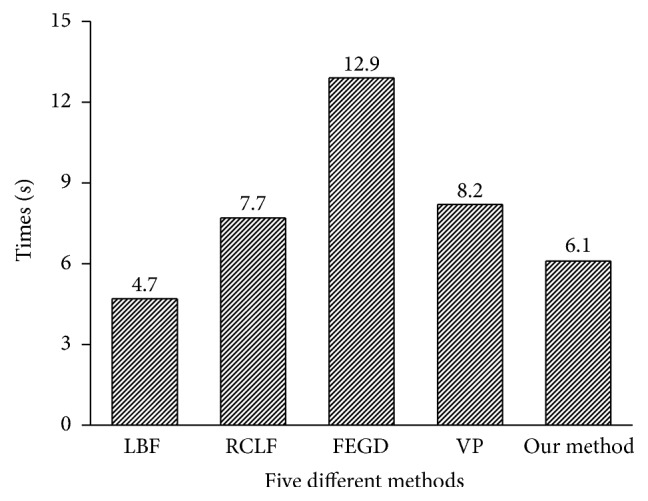
Average processing time for the five algorithms on a pulmonary nodule.

In addition to the above three metrics, the time complexity is also an important factor in assessing the performance of an algorithm.  Figure 8 shows the average processing time per nodule by our method and other three algorithms.

As can be seen from Figure 8, our method consumes less time than the RCLF, FEGD, and VP algorithms, but much more (1.4 s) than LBF algorithm. This is because that SUV information entropy of each point on the contour curve needs to be recalculated in each iteration. However, in terms of acceptable time complexity, we should pay more attention to the accuracy and stability of the segmentation algorithm, trying to avoid the errors of the lung cancer diagnosis caused by the incorrect nodule segmentation.

Accurate segmentation of juxta-vascular pulmonary nodules can improve the diagnostic accuracy rates of benign and malignant nodules to some extent. Our method, combining the metabolic and structural information of nodules, can drive the evolution of the initial contour curve. In aspect of DSC, Hausdorff FP, and consuming time metrics, the segmentation results of our method are superior to those of the other four methods.

## 6. Conclusion

In this paper, we present an automatic juxta-vascular nodules segmentation algorithm based on LBF active contour model in PET-CT images. In the method, SUV information entropy and the joint vector in PET-CT images are incorporated into the LBF model for driving the evolution of contour curve to stop at the boundary of the pulmonary nodule accurately. To verify the effectiveness of our method, we applied it on PET-CT images of 400 juxta-vascular nodules and the results were compared with the manual segmentation by an expert. Experimental results show that our method can obtain high-quality segmentation results and reduce the edge leakage to some extent. Compared with the existing methods, our method achieves better segmentation accuracy and stability. Thus, it is an accurate segmentation scheme for juxta-vascular pulmonary nodules. Because SUV information entropy of each point on contour curve needs to be recalculated in each iteration, it may consume more processing time. In future work, we need to find ways to optimize this issue.

## Figures and Tables

**Table 1:** Parameters settings in our method.

Index	Parameters	Value
1	*λ* in Section 3.1.2	0.1
2	*r* in Section 3.2.2	15
3	*σ* in Section 3.2.3	1.5
4	*ν*, *μ*, *χ*, *K*_max_ in Section 3.2.4	0.001*∗*255^2^, 1, 10^−3^, 300

The parameters of LBF, RCLF, FEGD, and VP are stetted according to their original paper.

**Table 2:** Average values of DSC, HD, and FP for five methods on pulmonary nodules.

Metrics	LBF	RCLF	FEGD	VP	Our method
DSC (%)	87.43	90.46	91.38	89.02	**92.35**
HD (mm)	2.87	2.40	2.43	2.58	**2.19**
FP (%)	6.16	4.30	4.67	4.80	**3.33**
